# Brachial Plexus Injury Following Fracture-Dislocation of the Proximal Humerus: A Case Report

**DOI:** 10.7759/cureus.72593

**Published:** 2024-10-28

**Authors:** Samuel J Smith, Andy Webb

**Affiliations:** 1 Anesthesia, Lincoln County Hospital, Lincoln, GBR

**Keywords:** brachial plexus block, brachial plexus injury, clinical electro-diagnostics, electromyography (emg), nerve conduction studies (ncs), orthopedics and traumatology, proximal humerus fracture-dislocation, regional anesthesiology, shoulder reduction

## Abstract

Fracture-dislocations of the proximal humerus with brachial plexus injury are exceedingly rare, and although infrequently encountered, it is important to recognize this complication due to its potentially devastating impact. We present the case of a 75-year-old female who sustained the described injury following a fall onto their left arm, demonstrating combined sensory and motor deficits in the radial, median, and ulnar distribution of the left forearm and hand shortly afterward. Immediate management involved closed reduction under anesthesia, resulting in the improvement of neurological symptoms. For definitive management, surgical fixation of the fracture-dislocation is normally recommended in cases with an associated brachial plexus injury. However, due to a range of personal factors, the patient decided against this. Physiotherapy plays an important role in the management of these injuries, aiding mobility and recovery, with another key part of the follow-up being electrodiagnostic studies. These allow the physician to assess the extent of injury, monitor recovery for prognostication, and aid decisions regarding further surgical management. The importance of these studies is highlighted by the finding of severe brachial plexus injury, despite minimal pathological changes on MRI. Rare but significant, it is important to maintain a high index of suspicion for these injuries and consider underlying risk factors, with prompt surgical input required to optimize outcomes. Alongside surgical management, a considered approach must be taken by the anesthetic team, as regional anesthesia can confound post-procedure neurological assessment.

## Introduction

Upper arm injuries are a frequent presentation to the emergency department, with proximal humerus fractures accounting for an estimated 5%-6% of adult fractures and anterior shoulder dislocations being the most common dislocation seen [1,2]. However, combined fracture-dislocations of the proximal humerus are rare and usually involve higher energy mechanisms of injury [3]. Due to the anatomy of the upper arm, a thorough neurovascular examination is an important part of assessing these injuries, with any associated deficit requiring urgent surgical input. Brachial plexus injury is a rare but significant complication of upper arm injuries, and it is pertinent to be mindful of this.

Although rarely encountered, fracture-dislocations of the proximal humerus with brachial plexus injury are complex and require careful surgical consideration for optimal patient outcomes. These injuries can lead to significant morbidity, with work by Landers et al. demonstrating a negative impact on psychological well-being [4]. Alarmingly, 33% of the cohort disclosed suicidal ideation and 19% met the criteria for post-traumatic stress disorder and depression. More recent work by Dorich et al. found that impacts on arm and hand movement, health and well-being, and career are commonly reported by patients [5]. This case report adds to the limited literature on the topic, providing education for clinicians on this less commonly encountered presentation. 

## Case presentation

**Figure 1:**
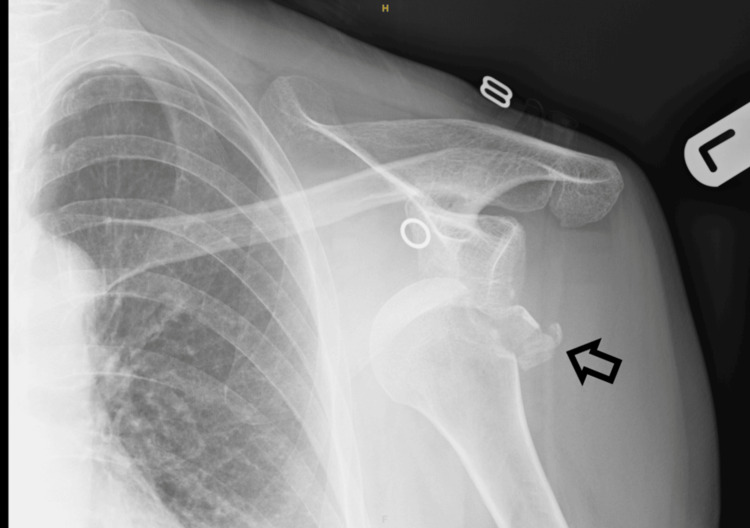
X-ray of the left shoulder in AP view demonstrates a fracture-dislocation of the proximal humerus

This case report describes a left proximal humerus fracture-dislocation with brachial plexus injury in a 75-year-old female patient following a fall. Neurological symptoms were described by the patient shortly following the injury, including evidence of multiple branch deficits. On examination, reduced sensation was present throughout the ulnar, median, and radial regions of the forearm and hand, alongside reduced muscle power and a prominent wrist drop. An X-ray of the left shoulder was performed (Figure 1), and the patient was admitted to the orthopedic team. Prompt closed reduction of the fracture-dislocation under anesthesia was performed with the aim of decompressing the brachial plexus, following which a sling was applied and regular neurological examinations were undertaken to monitor recovery. Expert opinion was sought and a discussion was had with the patient and chosen relatives regarding the recommendation for subsequent surgical fixation by an upper limb surgeon. However, due to a range of personal factors and preferences, the patient decided against this. 

**Figure 2:**
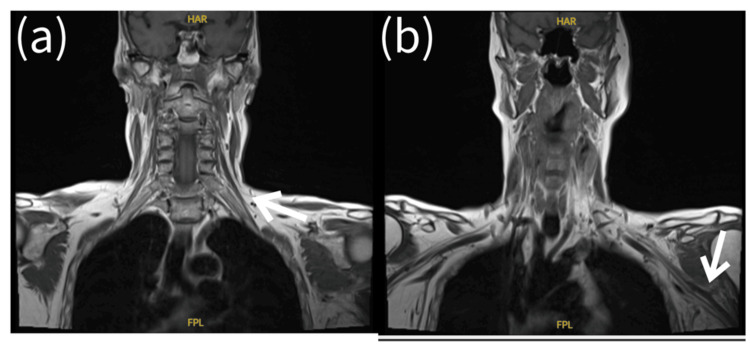
MRI brachial plexus findings (a) Demonstrates preserved nerve continuity in the left proximal brachial plexus. (b) Demonstrates edema around the left distal brachial plexus but preserved nerve continuity.

Significant improvement was noted in both the patients' subjective symptoms and on objective neurological examination in the days following the closed reduction, with regular physiotherapy input aiding recovery. By the time of discharge, the wrist drop had resolved and a sensation had improved. Whilst an inpatient, neurology review was arranged and an MRI was conducted, which found evidence of edema around the distal brachial plexus but preserved nerve continuity (Figure 2). On discharge, outpatient electrodiagnostic testing was arranged, which demonstrated severe left brachial plexus injury predominantly involving the medial and lateral cords (Tables 1, 2). This is an important part of the follow-up, aiding prognostication and helping to guide if any further surgical input is needed in the case of poor recovery. 

**Table 1:** Motor NCD Demonstrates attenuated left median and ulnar motor responses, with preserved left radial motor response. NR: non-recordable; NCD: nerve conduction studies

Site	Latency (ms)	Amplitude (mV)	Amplitude difference (%)	Duration (ms)	Duration difference (%)	Conduction velocity (m/s)
Left median (abductor pollicis brevis) motor - wrist	3.6	0.14	-	15.9	-	-
Left median (abductor pollicis brevis) motor - elbow	NR	NR	NR	NR	NR	NR
Left radial (extensor indicis proprius) motor - forearm	1.4	2.6	-	24.9	-	-
Left ulnar (abductor digiti minimi) motor - wrist	2.7	0.66	-	24.6	-	-
Left ulnar (abductor digiti minimi) motor - below elbow	6.4	0.57	-8	21.9	-11	56.8
Left ulnar (abductor digiti minimi) motor - above elbow	8.9	0.62	11	25.1	15	36

**Table 2:** Sensory NCD Demonstrates attenuated left median, ulnar, and antebrachial sensory responses, with preserved left radial sensory response. NR: non-recordable; NCD: nerve conduction studies

Site	Onset latency (ms)	Amplitude (mV)	Distance (ms)	Conduction velocity (m/s)
Left-hand sensory median: wrist - digit II	2.6	2.1	130	50
Left-hand sensory median: wrist - digit III	NR	NR	-	NR
Left-hand sensory ulnar: wrist - digit V	2.4	1.42	110	45.8
Left lateral antebrachial cutaneous sensory: lateral biceps - lateral forearm	1.23	1.13	80	65
Left medial antebrachial cutaneous sensory: elbow - medial forearm	NR	NR	-	NR
Left radial sensory: forearm - anatomical snuffbox	1.55	30.3	80	51.6

This case posed a dilemma for the anesthetic team and required careful consideration, with the usual practice being the administration of an upper limb block. This is used to either facilitate the procedure without general anesthesia or to provide accompanying analgesia. However, given the brachial plexus injury, this was avoided so that any neurological recovery could be monitored closely post-procedure. 

## Discussion

Brachial plexus injury following a proximal humerus fracture-dislocation is a rare presentation and raises interesting learning points. When assessing a patient, it is important to be mindful of underlying risk factors, with the described patient being at high risk for a fracture of the humerus due to being over the age of 60, female, and having osteoporosis [6,7]. Work by Robinson et al. found that following upper arm injury, complex neurological deficits are more commonly seen in patients aged 60 years or older, demonstrating the importance of careful consideration in this instance [8]. In cases of suspected proximal humerus fracture-dislocation, it is pertinent to assess neurovascular status, both by inquiry into the patient's history and by subsequent examination. Due to the significant pain caused by these injuries, the patient will immobilize the affected arm, meaning a high index of suspicion is needed by the clinician to reduce the chance of missing this complication. Given the associated neurological deficit, the surgical team recommended fixation of the proximal humerus fracture-dislocation, in conjunction with expert advice from the Royal Orthopaedic Hospital, following the initial closed reduction. As true for all of medicine, the role of the doctor here is to present recommendations based on the best evidence and expertise whilst considering the patient’s wishes, fears, and personal circumstances. After careful consideration of the risks and benefits, the patient made an informed decision to avoid operative management. 

As eluded to in the case presentation, careful consideration was needed by the anesthetics team regarding the use of regional techniques. Brachial plexus blocks (e.g., interscalene block or supraclavicular block) can be used as sole anesthesia or to accompany general anesthesia in upper limb procedures, improving the patient experience. Regarding interscalene blocks, a meta-analysis of 23 randomized controlled trials found reduced severity of pain at eight hours post-procedure, reduced opiate use up to 12 hours, and reduced time to discharge from the hospital when administered [9]. This is further consolidated by findings of improved Disability of the Arm, Shoulder, and Hand questionnaire (DASH) scores in patients receiving an interscalene block for upper arm procedures compared to those who did not [10]. This is to say that a balanced risk versus benefit analysis and discussion with the patient and surgical team are needed when formulating the anesthetic plan. In this instance, the confounding effect of regional anesthesia on post-operative neurological assessment was deemed unacceptable, and general anesthesia was performed without the use of regional techniques. 

Brachial plexus injuries can have a significant impact on quality of life by impacting a range of daily activities. Work by Bhooshan et al. found that disability following brachial plexus injury negatively impacted health-related quality of life measures, with the largest impact being seen in the psychological domain. Of the patient's follow-up, over half described neuropathic pain, and this was identified as one of the important factors negatively impacting the quality of life [11]. Given the deleterious impact, brachial plexus injury can have early recognition with appropriate management, and follow-up is vital, including the use of electrodiagnostic studies. A combination of nerve conduction studies (NCS) and electromyography (EMG) can be used by the physician to help localize and then prognosticate these injuries [12]. The importance of these tests is highlighted by the MRI showing minimal pathological changes despite severe injury. NCS test for both sensory nerve and compound muscle action potentials between two skin surface electrodes placed over the course of a nerve, therefore allowing for localization of a lesion (reduced action potential amplitude, increased latency, and reduced conduction velocity being considered pathological). EMG involves the insertion of a small needle into the muscle of interest to measure motor unit action potentials at rest and then on voluntary contraction, aiding in the assessment of neuronal injury.

## Conclusions

Fracture-dislocations of the proximal humerus with brachial plexus injury are exceedingly rare, having potentially devastating impacts on patients and requiring early recognition and surgical input. The importance of electrodiagnostic studies is highlighted by this case, with MRI showing minimal pathological changes despite severe injury. This case also required pause for thought by the anesthetics team in their approach to delivering care, with the confounding effect of regional anesthesia on the neurological assessment being deemed unacceptable and demonstrating the need to tailor anesthesia to the individual and their condition. It is hoped that this case report will promote learning around the topic and encourage clinicians to consider this rare but significant complication of upper limb injury. 

## References

[1] Court-Brown CM, Caesar B (2006). Epidemiology of adult fractures: a review. Injury.

[2] Abrams R, Akbarnia H (2024). Shoulder dislocations overview. StatPearls [internet].

[3] Labrum JT IV, Kuttner NP, Atwan Y, Sanchez-Sotelo J, Barlow JD (2023). Fracture dislocations of the glenohumeral joint. Curr Rev Musculoskelet Med.

[4] Landers ZA, Jethanandani R, Lee SK, Mancuso CA, Seehaus M, Wolfe SW (2018). The psychological impact of adult traumatic brachial plexus injury. J Hand Surg Am.

[5] Dorich JM, Whiting J, Plano Clark VL, Ittenbach RF, Cornwall R (2024). Impact of brachial plexus birth injury on health-related quality of life in adulthood: a mixed methods survey study. Disabil Rehabil.

[6] Launonen AP, Lepola V, Saranko A, Flinkkilä T, Laitinen M, Mattila VM (2015). Epidemiology of proximal humerus fractures. Arch Osteoporos.

[7] Bergdahl C, Ekholm C, Wennergren D, Nilsson F, Möller M (2016). Epidemiology and patho-anatomical pattern of 2,011 humeral fractures: data from the Swedish Fracture Register. BMC Musculoskelet Disord.

[8] Robinson CM, Shur N, Sharpe T, Ray A, Murray IR (2012). Injuries associated with traumatic anterior glenohumeral dislocations. J Bone Joint Surg Am.

[9] Abdallah FW, Halpern SH, Aoyama K, Brull R (2015). Will the real benefits of single-shot interscalene block please stand up? A systematic review and meta-analysis. Anesth Analg.

[10] Egol KA, Forman J, Ong C, Rosenberg A, Karia R, Zuckerman JD (2014). Regional anesthesia improves outcome in patients undergoing proximal humerus fracture repair. Bull Hosp Jt Dis.

[11] Bhooshan LS, Gopal VV, Baburaj PT (2023). Impact of disability in the quality of life of patients with traumatic brachial plexus injuries based on a questionnaire survey in a tertiary center in South India. J Neurosci Rural Pract.

[12] Antonovich D, Dua A (2024). Electrodiagnostic evaluation of brachial plexopathies. StatPearls [Internet].

